# Evolutionary Dynamics and Transboundary Spread of Senecavirus A: The Role of Recombination and Global Swine Trade

**DOI:** 10.1155/tbed/6312703

**Published:** 2026-10-19

**Authors:** Yeqiu Li, Yongqiu Cui, Xinyu Gao, Ying Ji, Penghui Zeng, Siting Li, Junnan Chen, Peng Hui, Jianwei Zhou, Dedong Wang, Jiawu Wan, Fanshu Ma, Lei Hou, Jue Liu

**Affiliations:** ^1^ College of Veterinary Medicine Yangzhou University Yangzhou China yzu.edu.cn; ^2^ Jiangsu Co-Innovation Center for Prevention and Control of Important Animal Infectious Diseases and Zoonoses Yangzhou University Yangzhou 225009 China yzu.edu.cn; ^3^ Jiangsu Interdisciplinary Center for Zoonoses and Biosafety Yangzhou University Yangzhou 225009 China yzu.edu.cn; ^4^ Jiangsu Key Laboratory of Zoonosis Yangzhou University Yangzhou 225009 China yzu.edu.cn

**Keywords:** genomic characterization, isolation and identification, phylogeography, recombinant, Senecavirus A (SVA)

## Abstract

Senecavirus A (SVA) is an emerging swine pathogen associated with vesicular disease and neonatal mortality, posing significant challenges to global swine health. Here, we report the isolation and identification of two contemporary SVA strains, JS01 and JS02, from clinically suspected cases in China. Both isolates demonstrated high replication competence and clustered within Clade Ib, the dominant globally circulating swine‐associated lineage, showing close genetic ties to strains from southeastern China. Phylogenetic and recombination analyses identified multiple putative recombination events within nonstructural genomic regions, suggesting that recombination drives SVA diversification. Spatiotemporal analysis revealed uneven sequence availability over time and across regions, with sampling concentrated in Asia and the Americas during 2015–2018. Phylogeographic reconstruction indicated supported geographic transition connecting Brazil to China, the United States, and Canada. Notably, the inferred dispersal pattern visually overlapped with global live‐swine trade networks, pointing to a potential possible link with animal trade. Furthermore, our analysis uncovered a divergent, marine‐associated lineage (Clade II) comprising Senecavirus‐like sequences from dolphins, which represents a distinct sister clade to terrestrial swine‐associated viruses. These findings highlight the evolutionary complexity of SVA and emphasize the importance of genomic surveillance for investigating viral dispersal and its relationship with animal movements.

## 1. Introduction

Senecavirus A (SVA) is a nonenveloped, single‐stranded positive‐sense RNA virus belonging to the *Picornaviridae* family and represents the sole species of the genus *Senecavirus* [1]. Phylogenetic analyses have confirmed its close evolutionary relationship with viruses of the genus *Cardioviruses*. SVA particles exhibit an icosahedral morphology with a diameter of 25–30 nm [2]. Electron microscopy reveals that purified SVA virions display a quasispherical or hexagonal appearance [3]. The full‐length SVA genome is ~7.2–7.3 kb in size and comprises a single open reading frame (ORF) that encodes a 2181‐amino‐acid polyprotein. This precursor polyprotein is proteolytically cleaved into a leader protein (L) and three precursor proteins (P1, P2, and P3). The P1 precursor encodes the structural proteins VP1, VP2, VP3, and VP4, which collectively assemble to form the viral capsid. Specifically, VP1, VP2, and VP3 are exposed on the outer surface of the capsid and contain major antigenic epitopes, whereas the smaller VP4 protein is located internally within the capsid. The P2 and P3 precursors give rise to non‐structural proteins, including 2A, 2B, 2C, 3A, 3B, 3C, and 3D [4–8].

SVA was first identified in swine populations in the United States in the 1980s, with subsequent detections in Canada, Brazil, and Colombia [9, 10]. In 2002, an SVA strain was accidentally isolated from contaminated adenovirus cultures propagated in human fetal retinal PER.C6 cells and was designated SVA‐001; however, subsequent pathogenicity assays demonstrated that this prototype strain exhibited negligible virulence [1]. In 2007, vesicular and ulcerative lesions emerged on the snouts and coronary bands of swine in specific regions of the United States, coinciding with the first confirmation of SVA infection in Canadian pig farms. By the end of 2014, SVA outbreaks had been documented in Brazil, and genomic analysis indicated that Brazilian SVA strains share 94.2%–96.5% nucleotide sequence homology with the prototype SVA‐001 strain. In 2016, SVA was detected in Colombia, with the identified strain displaying 98.5%–99% nucleotide identity to a previously reported US strain (GenBank accession: KX857728) [11]. In October 2016, vesicular disease cases consistent with SVA infection were reported in Thailand. Whole‐genome sequencing confirmed that the Thai SVA strain shares 98.2% nucleotide homology with a Canadian isolate (KX11‐55910‐3) [12, 13].

The first domestic SVA infection case in China was reported in Guangdong Province in April 2015 [14]. In 2016, SVA strains detected in Hubei Province shared high genetic homology with the 2015 Guangdong isolate, indicating a common epidemiological origin of these Chinese strains [15]. Meanwhile, an SVA outbreak occurred in Heilongjiang Province in the same year, and the Heilongjiang isolate exhibited high sequence similarity to prevalent US SVA strains [16]. Since then, SVA has spread widely across multiple Chinese provinces, including Hubei, Henan, and Heilongjiang, causing substantial economic losses to the domestic swine industry. Mutations have continued to accumulate in circulating strains [17–20]. Continuous genetic diversification has been observed in Chinese SVA isolates since the first identification in 2015, with genetically distinct variants circulating in different regional pig herds [21]. Phylogenetically, most Chinese SVA isolates cluster closely with US‐origin strains [22]. Moreover, natural recombination events have been frequently detected among circulating SVA strains in China [23–25].

SVA infection shows no clear seasonal pattern, is characterized by high morbidity but low mortality, and occurs predominantly as sporadic cases rather than large‐scale outbreaks [12, 26–28]. Notably, SVA‐neutralizing antibodies have been detected in serum samples from cattle and sheep, suggesting a potential cross‐species transmission risk. The incubation period of SVA is generally 4–5 days. The virus is capable of infecting pigs of all ages and breeds, with variable disease severity and mortality rates ranging from 30% to 70% [29].

In the present study, two SVA strains were successfully isolated and subjected to whole‐genome sequencing. Phylogenetic analysis was performed to clarify the genetic evolutionary relationships between the two novel isolates and previously reported SVA strains from China and other countries. Furthermore, comprehensive bioinformatics analyses were conducted to characterize the genotypes and key genetic mutations of circulating SVA strains.

## 2. Materials and Methods

### 2.1. Cells and Antibody

Baby hamster kidney cells (BHK‐21, ATCC CCL‐10) were cultured in Dulbecco’s Modified Eagle Medium (DMEM; Gibco, 11965092) supplemented with 10% fetal bovine serum (FBS; Gibco, 10099141), 100 U/mL penicillin, and 100 μg/mL streptomycin. Cell incubation was performed at 37°C under a humidified atmosphere with 5% CO_2_. The monoclonal antibody targeting the SVA VP1 protein was prepared and preserved in our laboratory for subsequent experimental use.

### 2.2. Sample Collection and Virus Isolation

Positive swine tissue samples used in this study were collected from farms in Hebei and Jiangsu, China, during 2016–2017, with the farm owners’ voluntary consent to donate them to us. As no clinical animal experiments were conducted, we did not apply for institutional animal ethics approval. Tissue samples were homogenized in sterile DMEM, and the resulting homogenates were clarified by low‐speed centrifugation, filtered through sterile 0.22 μm filters, and inoculated onto BHK‐21 cell monolayers. The cells were cultured at 37°C with 5% CO_2_ and monitored daily for cytopathic effects (CPEs) for up to 72 h. Cultures without evident CPEs were subjected to blind passage as necessary [15]. Successful viral isolation was confirmed via RT‐PCR, western blotting, and indirect immunofluorescence assay (IFA).

### 2.3. Plaque Purification

Virus‐containing supernatants were serially diluted and inoculated onto confluent BHK‐21 cell monolayers. Following viral adsorption, cells were overlaid with semisolid culture medium and incubated until discrete individual plaques were visible. Single plaques were carefully picked using sterile pipette tips and resuspended in DMEM containing 2% FBS. Plaque‐derived viruses were amplified in fresh BHK‐21 cells, and the purification procedure was repeated for three consecutive rounds to obtain homogeneous clonal viral stocks.

### 2.4. Virus Infection and Growth‐Curve Analysis

SVA stock viruses were propagated in BHK‐21 cells. Supernatants from cell cultures displaying typical CPEs were collected, clarified via centrifugation, and stored at −80°C for subsequent use. For multistep growth‐curve analysis, BHK‐21 cell monolayers were separately infected with the JS01 and JS02 isolates. Infected cell cultures were harvested at 6, 12, 24, 36, and 48 h postinfection (hpi). Viral titers were determined by serially diluting the harvested virus suspensions and inoculating the dilutions onto BHK‐21 cells. Titers were calculated using the Spearman–Kärber method and expressed as log_10_ TCID_50_/0.1 mL. The resulting viral titers were used to generate multistep growth curves.

### 2.5. Western Blotting

Proteins extracted from mock‐infected and JS01 (Jiangsu‐2016)‐ or JS02 (Hebei‐2017)‐infected BHK‐21 cells were quantified using a bicinchoninic acid (BCA) protein assay kit (Thermo Fisher Scientific, Cat. No. 23225). Equal amounts of protein were separated by SDS–PAGE and transferred onto nitrocellulose membranes (Pall, Cat. No. 66485). After blocking with 5% nonfat milk, the membranes were incubated with anti‐SVA VP1 and anti‐β‐actin primary antibodies, followed by the appropriate secondary antibodies. Protein bands were visualized using an AMERSHAM ImageQuant 800 chemiluminescence imaging system (GE Healthcare, USA).

### 2.6. Indirect IFA

BHK‐21 cells cultured overnight were mock‐infected or separately infected with the JS01 or JS02 isolate. At 12 hpi, the cells were fixed with 4% paraformaldehyde for 30 min, permeabilized with 0.1% Triton X‐100 (Sigma–Aldrich, T8787) for 10 min, and blocked with 5% nonfat milk at 37°C for 30 min. The cells were then incubated with the anti‐SVA VP1 primary antibody at 4°C overnight, followed by the appropriate fluorescent secondary antibody at 37°C for 1 h in the dark. The cells were counterstained with 4^′^,6‐diamidino‐2‐phenylindole (DAPI; Sigma–Aldrich, D8417). Fluorescence signals were examined using an immunofluorescence microscope (IX73; Olympus, Tokyo, Japan).

### 2.7. Whole‐Genome Sequencing

Viral RNA was extracted from clarified supernatants of SVA‐infected BHK‐21 cells using the TIANGEN Viral RNA Kit, and cDNA was synthesized with random primers using the RevertAid First Strand cDNA Synthesis Kit. Full‐length SVA genomes were amplified using overlapping PCR primers and subjected to bidirectional Sanger sequencing. Sequence reads were quality‐checked and assembled by alignment with representative SVA reference genomes. Ambiguous nucleotide sites were confirmed by repeated PCR amplification and sequencing.

### 2.8. Dataset Construction

A total of 288 complete SVA genome sequences were retrieved from GenBank on December 31, 2025, together with available collection dates, geographic locations, and host information (Table S1). Multiple sequence alignment was performed using a fast Fourier transform‐based algorithm, followed by manual refinement to correct alignment errors. Sequences with poor alignment quality, premature stop codons, or frameshift mutations were excluded to reduce potential bias in phylogenetic analyses [30]. The optimal nucleotide substitution model was selected based on Bayesian information criterion (BIC) scores using ModelFinder [31]. Bayes‐factor comparisons were used to select the molecular clock, demographic, and discrete‐trait substitution models [32]. A relaxed lognormal molecular clock [33], Bayesian skyline demographic model [34], and a symmetric model of transitions between discrete geographic states [35] were used in subsequent evolutionary analyses.

### 2.9. Recombination Detection

Potential recombination signals were initially screened using the PHI test in SplitsTree [36]. Datasets with statistically significant recombination signals (*p* < 0.05) were further analyzed using seven independent detection algorithms embedded in RDP4, including RDP, GENECONV, BootScan, MaxChi, Chimaera, SiScan and 3Seq [37–44]. Recombinant events were retained if supported by at least five methods with a multiple‐comparison‐corrected *p* value of <0.05. Similarity plots were used to assess changes in relative sequence similarity around inferred breakpoints. Parental‐like sequences represent the closest known matches in the dataset and should not be interpreted as experimentally confirmed direct parental strains. Putative recombinant sequences were excluded from downstream analyses, and no independent selection analysis was performed for these regions.

### 2.10. Phylogenetic Reconstruction

Phylogenetic signal in the curated nonrecombinant dataset was evaluated by likelihood‐mapping in IQ‐TREE (v1.6.12) and MEGA 7.0 [45, 46]. Nucleotide substitution saturation was assessed via Xia’s test in DAMBE (v7.3.5) by comparing the observed substitution saturation index (Iss) with the critical value (Iss.c). A significantly lower Iss value indicated negligible substitution saturation, confirming sufficient phylogenetic signal for tree reconstruction. Maximum‐likelihood (ML) phylogenetic trees were constructed using the optimal nucleotide substitution model screened previously. Phylogenetic clades were classified according to the established SVA evolutionary classification system, with all isolates assigned to porcine‐associated Clade Ia and Clade Ib based on tree topology [4, 25, 47]. Phylogenetic trees were visualized using FigTree (v1.4.4) and the interactive tree of life (iTOL).

### 2.11. Phylogeographic Analysis

Bayesian tip‐dated coalescent analyses were performed using BEAST (v1.10.4) to estimate the evolutionary rate, divergence time, and historical population dynamics of SVA [35, 48]. An uncorrelated lognormal relaxed molecular clock and the GTR+F+I+G4 model were applied to account for nucleotide rate heterogeneity. Three independent Markov chain Monte Carlo (MCMC) simulations were run, each with 3 × 10^8^ generations and sampling every 10,000 generations. The first 10% of MCMC samples were discarded as burn‐in, and model convergence was assessed using Tracer (v1.6; http://tree.bio.ed.ac.uk/software/tracer/). Parameters with an effective sample size (ESS) >200 were considered statistically reliable. All evolutionary parameters were estimated based on median values and 95% highest posterior density (HPD) intervals.

Temporal signal was evaluated using Bayesian evaluation of temporal signal (BETS) by comparing heterochronous and isochronous models through generalized stepping‐stone sampling, with log Bayes factor (BF) ≥ 5 considered evidence of temporal signal. Bayesian stochastic search variable selection (BSSVS) was used to identify supported geographic transition links [35]. Support for geographic transition links was classified as positive (BF > 3), strong (BF > 5), or very strong (BF > 10). Phylogeographic trees were visualized using FigTree (v1.4.4) and iTOL. Here, migration denotes inferred transitions between sampled geographic states rather than documented transmission events or movements of infected animals.

### 2.12. Spatiotemporal and Trade‐Associated Visualization Analysis

SVA collection dates and geographic coordinates were obtained from GenBank and manually verified. Month‐level records were aggregated into a continuous year–month matrix for seasonal‐trend decomposition using locally weighted regression and monthly heatmap visualization, whereas annual records were used for global mapping by collection year. All spatiotemporal distributions represent sequence availability trends indexed by collection date and location rather than direct measures of disease incidence or epidemic intensity.

Genomic distances and inferred geographic transitions were analyzed to characterize SVA dispersal patterns. Pairwise nucleotide distances were calculated from full‐genome alignments (excluding ambiguous sites and gaps) to construct a divergence heatmap comparing intra‐ and interregional variation. Posterior mean Markov jump counts summarized the inferred numbers of transitions between sampled geographic states. Geographic transition links with a BF > 3 and posterior probability (PP) > 0.3 were retained for presentation.

To visually compare inferred geographic dispersal with international live‐swine trade patterns, global live swine trade data (2013–2024) were retrieved from the UN Comtrade Database (HS code 0103). Trade volumes were standardized to metric tons, prioritizing global‐level records to prevent double‐counting. Global import/export distribution maps were generated based on annual volumes, with supported geographic transition links overlaid to assess spatial concordance. All analyses and visualizations were conducted using customized R and Python scripts. Trade records reflect animal movements, whereas the overlaid phylogeographic links represent inferred geographic transitions. The comparison was descriptive, and no formal statistical association between pairwise viral migration rates and trade volumes was tested.

### 2.13. Statistical Analysis

Statistical analyses were performed using GraphPad Prism version 7.0 (GraphPad Software, San Diego, CA, USA). Viral titers obtained from three independent experiments were log_10_‐transformed and expressed as log_10_TCID_50_/0.1 mL. Data are presented as the mean ± standard deviation (SD). Statistical significance was determined using Student’s *t*‐test, with *p* < 0.05 considered statistically significant.

## 3. Results

### 3.1. Virus Isolation, Sequencing, and Phylogenetic Analysis

During long‐term surveillance of SVA in swine populations, two SVA‐positive tissue samples were identified from clinically suspected cases and subsequently subjected to virus isolation. Following inoculation of tissue homogenates onto BHK‐21 cells, characteristic CPEs, including cell shrinkage, rounding, increased cellular refractility, and disruption of the normal cell monolayer, were observed within 72 h. Two infectious SVA isolates were successfully rescued and designated JS01 and JS02.

To confirm successful virus isolation, an indirect IFA was performed using an anti‐SVA VP1 antibody. Distinct cytoplasmic fluorescence signals specific to VP1 were detected in JS01‐ and JS02‐infected cells, whereas no specific fluorescence was observed in mock‐infected cells. Cell nuclei were visualized via DAPI staining (Figure 1A). Western blotting further identified protein bands corresponding to the expected VP1 molecular weight in JS01‐ and JS02‐infected cell lysates, with no equivalent band present in mock‐infected controls (Figure 1B). Together, these findings confirmed successful isolation of the two SVA strains. Multistep growth‐curve analysis demonstrated that both JS01 and JS02 replicated efficiently in BHK‐21 cells: viral titers increased rapidly postinfection and reached peak levels at 36 hpi (Figure 1C).

**Figure 1:**
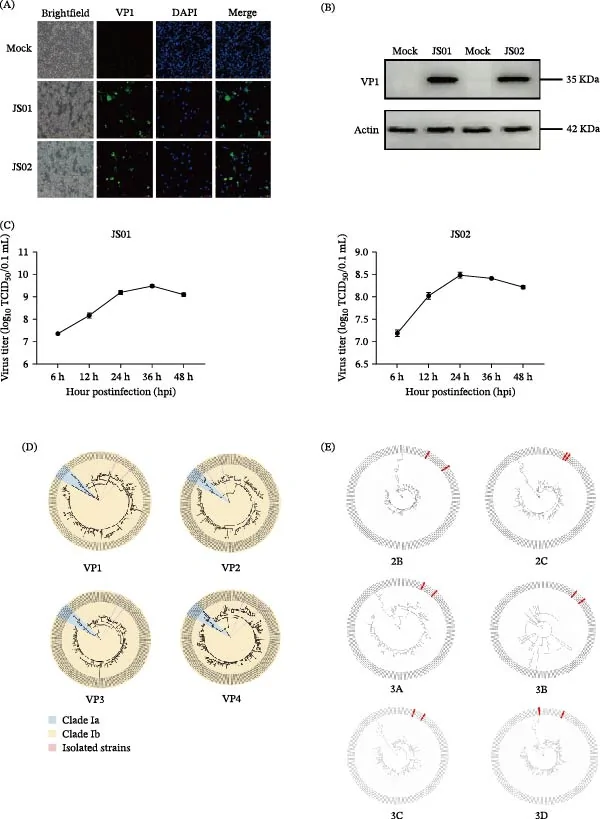
Isolation, identification, growth kinetics, and phylogenetic characterization of Senecavirus A (SVA) strains JS01 and JS02. (A) Immunofluorescence assay detecting viral VP1 protein expression in cells infected with JS01 or JS02 strains. Green fluorescence indicates VP1‐positive infected cells, and blue fluorescence represents DAPI‐stained cell nuclei. Mock‐infected cells were used as the negative control. (B) Western blot analysis of VP1 protein expression in JS01‐ and JS02‐infected cells. (C) Growth kinetics of JS01 and JS02 isolates at 6, 12, 24, 36, and 48 h postinfection (hpi). Data are presented as the mean ± standard deviation (SD). (D) Phylogenetic analysis based on the structural protein‐coding genes (VP1, VP2, VP3, and VP4). Different clades are distinguished by distinct background colors, and the two isolated SVA strains are highlighted. (E) Phylogenetic analysis based on the nonstructural protein‐coding genes (2B, 2C, 3A, 3B, 3C, and 3D), with the isolated JS01 and JS02 strains marked in red.

Whole‐genome sequencing revealed genome lengths of 7268 bp for JS01 and 7265 bp for JS02. Both isolates exhibited canonical SVA genome organization and lacked premature stop codons or frameshift mutations within coding regions. Pairwise sequence comparisons indicated that the two isolates shared up to 99% nucleotide identity with representative SVA strains deposited in the NCBI GenBank database, confirming JS01 and JS02 as contemporary swine‐origin SVA strains (Table S1).

To resolve the evolutionary placement of the two isolates, phylogenetic reconstructions were generated using representative SVA sequences retrieved from GenBank. Following sequence alignment, model selection, and ML tree construction, the analyzed SVA strains segregated into two major swine‐associated clades. Clade Ia primarily comprised earlier epidemic strains, whereas Clade Ib contained the majority of globally circulating contemporary strains. Both JS01 and JS02 clustered robustly within Clade Ib, demonstrating that these isolates belong to the currently prevailing swine‐associated SVA lineage (Figure 1D).

Gene‐wise phylogenetic analysis further indicated that JS01 and JS02 were closely related to previously reported Chinese SVA strains, in particular variants originating from Guangdong and Jiangsu Provinces. For JS01, the 2 B, 2 C, 3 A, 3 C, 3D, VP1, and VP3 genes shared the highest sequence similarity with strain KX173340, while the 3 B, VP2, and VP4 genes clustered separately with KX713137, MG765566, and KX173338, respectively. For JS02, the structural genes VP1, VP2, VP3, and VP4, along with the nonstructural genes 2B, 2C, 3A, and 3D, were most closely related to MG765560, whereas 3B and 3C grouped with KX173339 and MK170056, respectively (Figure 1D,E). Notably, most of these closely related reference strains were isolated in Guangzhou, Guangdong Province, with additional related sequences originating from Yangzhou, Jiangsu Province. Collectively, these data suggest that JS01 and JS02 are phylogenetically affiliated with SVA strains circulating in southeast China and are consistent with local circulation of Clade Ib viruses.

### 3.2. Detection of Putative Recombination Events

Recombination events may confound computational analyses used for epidemiological traceability. To eliminate bias introduced by recombinant sequences in downstream analyses, putative recombinant strains were excluded from the main dataset. We implemented RDP4 software to screen for recombination signals using complete genome sequences. Three putative recombination events were identified among the analyzed strains: (1) MH316113 was identified as a putative recombinant, with SVV/CHN/07/2017 (GenBank: MG765556) and CH‐LX‐01−2016 (GenBank: KX751945), identified as the closest known major‐ and minor‐parent‐like sequences, respectively. The inferred breakpoint positions were 5100–6800 (Figure 2A). (2) MF189001 was identified as a putative recombinant, with CH‐GD‐2017−01 (GenBank: MF189000) and CH‐FJ‐2017 (GenBank: KY747510) identified as the closest known major‐ and minor‐parent‐like sequences, with the recombinant interval located at positions 3800–5100 (Figure 2B). (3) MH064436 was identified as a putative recombinant, with USA/IN_Purdue_3698/2016 (GenBank: KY618837) and HB‐CH‐2016 (GenBank: KX377924) identified as the closest known major‐ and minor‐parent‐like sequences, respectively. The inferred breakpoint positions were 6200–6600 (Figure 2C). These putative recombination events suggest genetic exchange among SVA lineages represented in the sampled dataset.

**Figure 2:**
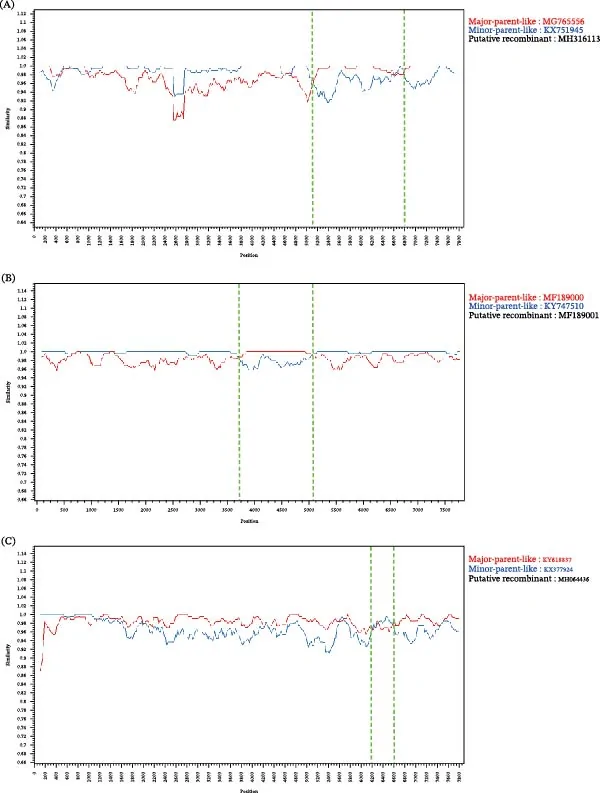
Similarity plot analysis of putative recombination events in Senecavirus A genomes. Similarity plots were used to assess changes in relative sequence similarity around the inferred recombination breakpoints. The *x*‐axis represents the genomic nucleotide position, and the *y*‐axis indicates the pairwise sequence similarity. The red and blue curves denote sequence similarity to the two corresponding reference sequences. Green dashed vertical lines mark the approximate inferred breakpoint positions. (A) Recombination analysis of putative recombinant MH316113, with MG765556 and KX751945 identified as the closest known major‐ and minor‐parent‐like sequences, respectively. (B) Recombination analysis of putative recombinant MF189001, with MF189000 and KY747510 identified as the closest known major‐ and minor‐parent‐like sequences, respectively. (C) Recombination analysis of putative recombinant MH064436, with KY618837 and KX377924 identified as the closest known major‐ and minor‐parent‐like sequences, respectively.

### 3.3. Temporal and Geographic Patterns of SVA Sequence Availability

Analysis of SVA‐positive sequence records revealed complex temporal and geographic patterns of sequence availability (Figure 3). The year–month heatmap (Figure 3A) showed that most SVA sequence records were dated between 2015 and 2018, with a pronounced sequence sampling peak in mid‐2017, reflecting a concentration of available sequences during this period. Sparse sequence records outside this time window suggest either possible low‐level circulation prior to widespread recognition of SVA or under‐sampling in earlier years.

**Figure 3:**
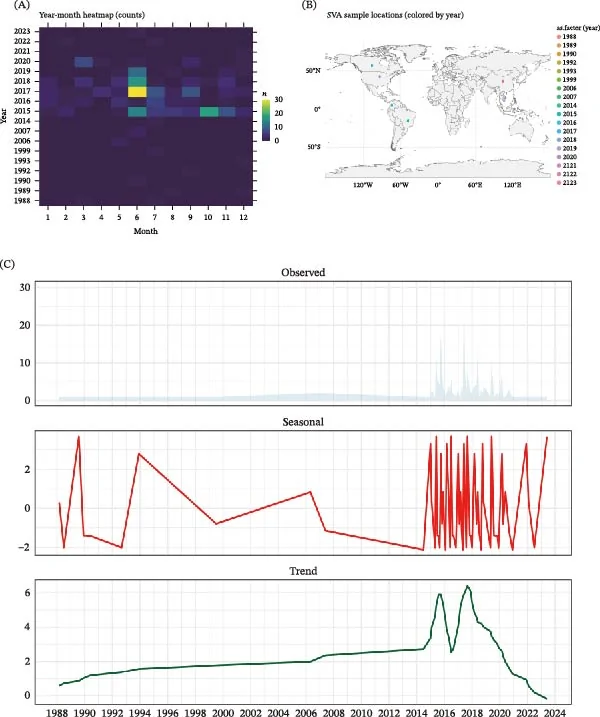
Temporal and geographical distribution of Senecavirus A (SVA) sequences used in this study. (A) Year‐month heatmap illustrating the temporal distribution of collected SVA sequences, with color intensity corresponding to the number of sequences obtained per month. (B) Global geographical distribution of SVA sampling sites, with sampling points colored according to the collection year. (C) Time‐series decomposition of SVA sequence counts, presenting the observed sequence distribution, seasonal variation component, and long‐term trend in sequence availability. The trend curve shows that the availability of SVA sequences increased after 2015 and gradually declined after 2018.

Geographic mapping (Figure 3B) demonstrated a broad yet uneven global distribution of sequence records spanning Asia, North and South America, and Europe. Sequence records were markedly concentrated in Asia, especially within China. This concentration identifies areas with greater sequence availability rather than establishing endemic foci or regions supporting sustained viral amplification. SVA detection patterns varied across regions, indicating heterogeneous regional dynamics rather than a synchronized global pattern. However, the underlying epidemiological processes driving these differences could not be resolved from sequence data alone.

Time‐series decomposition (Figure 3C) provided further insight into SVA sequence availability over time. Observed sequence counts reflected intermittent, sporadic detection events, while seasonal decomposition identified irregular mid‐year sequence‐count peaks. The long‐term trend component indicated a gradual rise in SVA sequence counts starting in the early 1990s, a peak during 2015–2018, and a subsequent decline. Taken together, these analyses demonstrate that the available SVA sequence dataset is characterized by episodic temporal clustering, variation in monthly sampling, and substantial geographic heterogeneity. These findings highlight the value of sustained global surveillance to characterize geographic and temporal concentrations of SVA sequence detections, laying a foundation for unraveling SVA ecology and supporting risk assessments for potential cross‐species transmission.

### 3.4. Evolutionary Dynamics of SVA

To further characterize the evolutionary dynamics of SVA, we incorporated the most recently available SVA sequences into phylogenetic analyses. Notably, phylogenies revealed clear host‐associated population structure within the global SVA dataset (Figure 4A). The majority of sequences fell within swine‐associated SVA lineages, subdivided into the previously defined Clades Ia and Ib. Clade Ib accommodated most contemporary swine‐origin strains, representing the dominant swine‐associated lineage in our dataset. In contrast, a small, well‐separated external branch, designated Clade II, was identified outside Clades Ia and Ib. This clade exclusively contained three publicly available Senecavirus‐like sequences recovered from dolphin (Delphinidae) liver samples, indicating the existence of a divergent marine‐associated lineage within global Senecavirus records. The unambiguous phylogenetic separation of Clade II from swine‐associated Clades Ia and Ib suggests that these dolphin‐derived sequences constitute a distinct sister lineage, rather than a subclade within known porcine SVA diversity.

**Figure 4:**
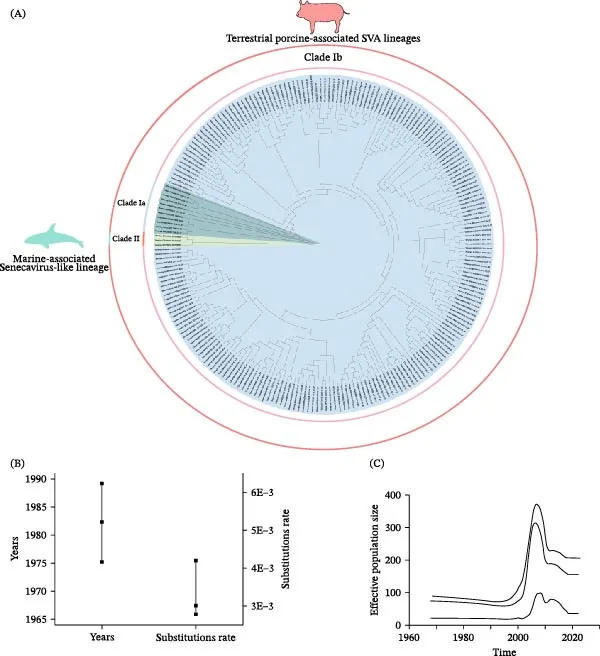
Phylogenetic structure and evolutionary dynamics of global Senecavirus A (SVA) populations. (A) Circular phylogenetic tree revealing the genetic relationships among representative Senecavirus‐related sequences. All strains are classified into three major lineages (Clade Ia, Clade Ib, and Clade II), and host silhouettes indicate the corresponding host association of each lineage. (B) Estimated evolutionary parameters of SVA, including the time to the most recent common ancestor (tMRCA) and genome‐wide nucleotide substitution rate. Dots represent mean estimated values, and vertical bars indicate 95% highest posterior density (HPD) intervals. (C) Bayesian skyline plot (BSP) showing the temporal dynamics of SVA effective population size. The central black curve indicates the median estimate, and the upper and lower boundary curves represent the 95% HPD intervals.

The mean genome‐wide evolutionary rate was estimated at 3.012 × 10^−3^ nucleotide substitutions per site per year (subs site^−1^ year^−1^), with a 95% HPD interval of 2.782 × 10^−3^ – 4.198 × 10^−3^ subs site^−1^ year^−1^. The time to the most recent common ancestor (tMRCA) of SVA was dated to 1982.338 (95% HPD: 1975.218–1989.203) (Figure 4B). Furthermore, the estimated viral effective population size increased rapidly in the early 21st century, peaked around 2010, and subsequently declined sharply (Figure 4C).

### 3.5. Phylogeographic Reconstruction of SVA Dispersal

Geographic transition links meeting the specified support thresholds (BF > 3 and PP > 0.3) were retained for interpretation. The reconstructed network connected sampled geographic states in Asia and the Americas (Figure 5; Table 1). Supported links connected Brazil with China (BF = 273.6984, PP = 0.933494), the United States (BF = 434.4746, PP = 0.96134), and Canada (BF = 824.5, PP = 1). These results indicate supported geographic connectivity involving Brazil within the sampled dataset.

**Figure 5:**
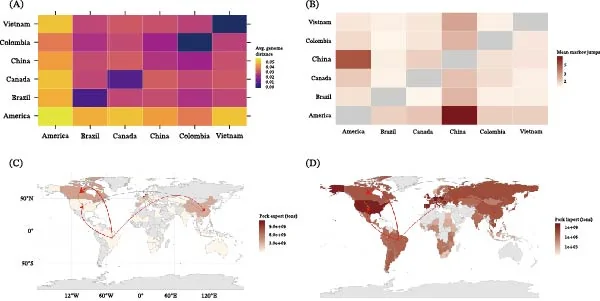
Phylogeographic diffusion of Senecavirus A (SVA) and comparison with global live swine trade. (A) Heatmap of average genomic distances among SVA strains from different countries and regions. The color gradient represents pairwise average nucleotide distances, with warmer colors indicating higher genomic divergence. (B) Heatmap of posterior mean Markov jumps derived from phylogeographic reconstruction, reflecting the relative frequency of SVA migration events across geographical regions. Warmer colors indicate higher inferred transition counts. (C) Global distribution of live swine export volumes overlaid with inferred SVA migration routes. Background shading indicates country‐level live swine export volumes (metric tons), calculated based on UN Comtrade/ComtradePlus records under HS code 0103. (D) Global distribution of live swine import volumes overlaid with inferred SVA migration routes. Background shading represents country‐level live swine import volumes (metric tons). Curved arrows indicate statistically supported SVA migration pathways between countries/regions, with only routes with Bayes factor (BF) > 3 and posterior probability (PP) > 0.3 displayed.

**Table 1:** Geographic transition links supported by Bayesian phylogeographic analysis.

Origin	Destination	Bayes factor (BF > 3)	Posterior probability (PP > 0.3)
Brazil	Canada	824.5	1
Brazil	America	434.4746	0.96134
Brazil	China	273.6984	0.933494
Canada	America	87.1343	0.914697
America	China	47.65254	0.891544

*Note*: Links were inferred using discrete‐trait phylogeographic analysis. Only links with a BF > 3 and PP > 0.3 are presented. These links represent statistical support for geographic connectivity and should not be interpreted as documented transmission events.

To explore the potential concordance between SVA phylogeography and international live‐swine trade, we integrated four complementary descriptive modules examining viral genetic relatedness, inferred geographic‐state transitions, and trade patterns (Figure 5). The pairwise genomic distance heatmap revealed varying degrees of genetic relatedness among SVA sequences from different countries and regions (Figure 5A). The posterior mean Markov jump heatmap indicated high frequencies of inferred geographic transitions, particularly involving the United States, China, and Brazil (Figure 5B). These phylogeographic patterns were subsequently compared visually with the available trade data. Any observed concordance is exploratory and does not establish direct transmission or causal trade‐mediated dissemination of SVA.

Live swine trade mapping identified Brazil, the United States, and Canada as major export hubs, with commodity flows directed toward China, Vietnam, and other Asian markets (Figure 5C). Visual comparison of international live‐swine trade maps with the distribution of available SVA sequences showed spatial overlap between regions with high recorded swine‐import volumes and regions with relatively concentrated SVA sequence records after 2015 (Figure 5D).

Collectively, these analyses revealed spatial concordance between global live‐swine trade patterns and inferred transboundary SVA geographic transitions. Several long‐distance trade corridors overlapped with supported phylogeographic links, while regions with relatively high recorded import or export volumes also contained concentrated SVA sequence records. These observations suggest a possible contribution of international live‐swine trade to the geographic dispersal of SVA; however, this exploratory comparison does not establish a statistical association or a direct causal relationship.

## 4. Discussion

In the present study, we recovered two SVA isolates from long‐term surveillance of swine populations and characterized their genetic and biological properties. These isolates replicated efficiently in cell culture and exhibited the canonical genomic organization of SVA, confirming that circulating strains remain replication‐competent. Phylogenetic analysis clustered both isolates within Clade Ib, the predominant porcine‐associated lineage, revealing a close genetic relationship to field isolates previously reported in Guangdong and Jiangsu provinces. These findings suggest that Clade Ib viruses continue to circulate in China and underscore the necessity of long‐term surveillance for detecting locally maintained variants.

To elucidate the evolutionary mechanisms driving this diversification, we examined genome‐wide phylogenetic relationships and recombination patterns. Gene‐by‐gene phylogenetic patterns for isolates JS01 and JS02 suggest that SVA evolution involves processes beyond simple clonal expansion. The mosaic relatedness observed across genomic regions is consistent with recombination contributing to lineage diversification [24, 49, 50]. The three putative recombination events identified here highlight the contribution of recombination to SVA genetic diversity and underscore the value of whole‐genome analysis [47, 51, 52]. These regions were not subjected to independent selection analysis, and their functional or adaptive significance remains unclear. Breakpoints were identified computationally, lacking independent read‐level or experimental validation.

The estimated evolutionary rate and tMRCA fall within the broad range reported in previous SVA studies [25, 51]. Although this agreement provides useful comparative context, these estimates remain conditional on the sampling timescale and molecular‐clock assumptions. Time‐dependent rate effects and undetected recombination may influence the inferred timescale, and sensitivity to the exclusion of specific genomic regions was not assessed. Furthermore, limited independent sampling of the marine‐associated lineage constrains robust lineage‐specific clock inference.

Epidemiologically, recombination signals among geographically distant strains indicate that SVA evolution is shaped by interconnected viral populations rather than isolated local circulation. Taken together, our findings demonstrate that SVA dispersal is geographically structured and that several supported phylogeographic links show spatial concordance with major international live‐swine trade patterns, within a broader context of regional ecological and epidemiological heterogeneity. Phylogeographic reconstruction placed Brazil in a central position within the sampled SVA geographic‐transition network, with strongly supported links to China, the United States, and Canada. Combined with Brazil’s prominent role in international live‐swine trade and export networks, this network position highlights Brazil as an epidemiologically important node for understanding and monitoring transboundary SVA dispersal (Figure 5) [53–56]. The spatial overlap between international live‐swine trade corridors and inferred SVA geographic‐transition links contextualizes these phylogeographic findings within cross‐border animal movements. This concordance provides a rationale for integrating trade information into SVA surveillance and prioritizing genomic sampling along major international live‐swine trade routes [57, 58]. However, the observed spatial concordance may also reflect geographical proximity and uneven sampling intensity, neither of which was formally accounted for in the trade comparison; it therefore motivates a hypothesis for further testing rather than establishing an independent association between trade and viral dispersal.

This pattern mirrors pathways proposed for other swine viruses, such as PEDV, involving transportation or contaminated feed ingredients [59–61]. In this context, the reconstructed SVA links reflect geographic connectivity among sampled populations rather than direct transmission chains between individual locations.

Simultaneously, temporal decomposition revealed sporadic but seasonally modulated sequence counts, with increased sampling frequency around mid‐year. Independent epidemiological evidence is required before attributing these patterns to environmental conditions, herd management practices, and host population dynamics [2, 62]. Differences in surveillance capacity, research funding, and sequence‐submission practices may bias the temporal and geographic representation of GenBank records; therefore, the observed sampling peaks and regional concentrations should not be interpreted as direct measures of disease incidence or demographic expansion. The high effective viral population size observed in China likely reflects sustained local circulation and ongoing genetic diversification, driven by dense swine populations, heterogeneous farming systems, and variable biosecurity practices [63]. A comparable epidemiological context has been described for African swine fever virus in China, where backyard and small‐scale farms contributed to viral maintenance and onward spread [64]. Taken together, these observations support a conceptual framework for interpreting SVA persistence and geographic expansion that incorporates local circulation and diversification with the potential contribution of live‐pig movements and trade networks to long‐distance dispersal [53, 62]. From a control perspective, these findings underscore the importance of integrated intervention strategies, including strengthened biosecurity regarding international animal movement, real‐time genomic surveillance, and improved on‐farm prevention in high‐risk settings [59, 65].

Beyond domestic swine populations, the identification of dolphin‐associated Senecavirus‐like sequences broadens the phylogenetic diversity represented in the available Senecavirus sequence records. Clade II formed a distinct sister lineage to terrestrial Clades Ia/Ib. Its separation from sampled swine‐associated lineages highlights a component of Senecavirus diversity that is not captured by surveillance focused exclusively on domestic pigs. This pattern supports the hypothesis that marine‐associated environments harbor additional, undersampled Senecavirus‐related diversity, providing a rationale for targeted investigation of marine hosts and their surrounding habitats (Figure 4A) [66, 67].

Although direct ecological links remain to be confirmed, agricultural runoff, waste discharge, and indirect contact among animals sharing coastal habitats may increase opportunities for contact between terrestrial and marine microbial communities. These possibilities constitute hypotheses for investigating the ecological context of the marine‐associated sequences. Studies of influenza A viruses in wild birds illustrate the importance of host ecology in viral distribution [68]. The recovery of Senecavirus‐like sequences from liver‐derived samples raises questions regarding tissue association, highlighting the value of combining molecular detection with pathological investigation, as illustrated by research on cetacean morbillivirus [69]. Given the environmental persistence reported for several nonenveloped picornaviruses, the survival and infectivity of Senecavirus‐related viruses in aquatic environments warrant investigation [70]. Climate‐related environmental variation may also influence the proposed coastal ecological connections, as suggested by observations in other aquatic pathogens [71]. Therefore, integrated surveillance of coastal swine farms, surrounding water systems, and wild marine mammals would provide a framework for testing these ecological connections and characterizing the distribution of Senecavirus‐related diversity [72, 73].

## 5. Conclusion

This study characterizes two contemporary Clade Ib SVA isolates from China and integrates evidence of recombination, genetic diversification, and inferred geographic dispersal to advance understanding of SVA evolution and transboundary connectivity. The spatial concordance between inferred geographic transitions and live‐swine trade patterns underscores the importance of incorporating animal movement data into genomic surveillance strategies. Additionally, the inclusion of dolphin‐associated Senecavirus‐like sequences reveals a divergent lineage and expands the sequence diversity represented in the analysis. Collectively, these findings refine the understanding of SVA genetic diversity and dispersal dynamics and inform priorities for genomic surveillance in swine populations.

## Author Contributions

**Yeqiu Li**: investigation, methodology, formal analysis, data curation, visualization, writing – original draft. **Yongqiu Cui**: investigation, methodology, virus isolation, validation, writing – original draft. **Xinyu Gao**: bioinformatics analysis, phylogenetic analysis, data curation, visualization. **Ying Ji and Penghui Zeng**: investigation, sample collection, validation, data curation. **Siting Li, Junnan Chen, and Peng Hui**: validation, formal analysis. **Jianwei Zhou, Dedong Wang, Jiawu Wan, and Fanshu Ma**: validation and manuscript review. **Lei Hou**: supervision, resources, writing – review and editing. **Jue Liu**: conceptualization, funding acquisition, project administration, supervision, writing – review and editing.

## Funding

This work was supported by the National Natural Science Foundation of China (Grants 32430105 and 32372975), the Introduction Program of High‐Level Innovation and Entrepreneurship Talents in Jiangsu Province, 111 Project D18007, and the Priority Academic Program Development of Jiangsu Higher Education Institutions (PAPD).

## Disclosure

All authors have read and agreed to the published version of the manuscript.

## Conflicts of Interest

The authors declare no conflicts of interest.

## Supporting Information

Additional supporting information can be found online in the Supporting Information section.

## Supporting information


**Supporting Information** Table S1: GenBank accession numbers and associated metadata for the Senecavirus A (SVA) sequences assessed in this study, including geographic origin (country/region), sampling date, host, and whether each sequence was included in or excluded from the BEAST analysis.

## Data Availability

The datasets used and analyzed in this study are provided within the article and its accompanying Supporting Information.
