# Supplementary material for: Evolutionary Dynamics and Transboundary Spread of Senecavirus A: The Role of Recombination and Global Swine Trade
Source: Transbound Emerg Dis. 2026 Oct 19;2026:6312703. doi: 10.1155/tbed/6312703 (PMC13678848; doi:10.1155/tbed/6312703)
Supplement: Supplementary file 1 [file TBED-2026-6312703-s001.docx]

Table S1 Information on Senecavirus A sequences

| Accession no. | Geographic states (country and region) | Sampling dates | Host | BEAST analysis inclusion status |
| --- | --- | --- | --- | --- |
| DQ641257 | USA | Not available | Not available | Included |
| KC667560 | Canada | 02-Nov-2011 | swine | Included |
| KR063107 | Brazil | 01-Feb-2015 | pig | Included |
| KR063108 | Brazil | 01-Feb-2015 | pig | Included |
| KR063109 | Brazil | 01-Feb-2015 | pig | Included |
| KT321458 | China | May-2015 | swine | Included |
| KT757280 | USA: Iowa | 23-Jul-2015 | swine | Included |
| KT757281 | USA: South Dakota | 31-Jul-2015 | swine | Included |
| KT757282 | USA: Iowa | 25-Aug-2015 | swine | Included |
| KU058182 | USA: Ohio | 19-Oct-2015 | pig | Included |
| KU058183 | USA: Ohio | 19-Oct-2015 | pig | Included |
| KU359210 | USA | 10-Oct-2015 | Not available | Included |
| KU359211 | USA | 10-Oct-2015 | Not available | Included |
| KU359212 | USA | 10-Oct-2015 | Not available | Included |
| KU359213 | USA | 10-Oct-2015 | swine | Included |
| KU359214 | USA | 10-Oct-2015 | mouse | Included |
| KU954086 | USA | Not available | swine | Included |
| KU954087 | USA: Iowa | 21-Jul-2015 | swine | Included |
| KU954088 | USA: Iowa | 22-Jul-2015 | swine | Included |
| KU954089 | USA: Iowa | 17-Aug-2015 | swine | Included |
| KU954090 | USA: Iowa | 17-Aug-2015 | swine | Included |
| KX019804 | USA | 2015 | porcine | Included |
| KX173338 | China | Oct-2015 | swine | Included |
| KX173339 | China | Oct-2015 | swine | Included |
| KX173340 | China | Nov-2015 | swine | Included |
| KX223836 | USA: Indiana | 08-Oct-2015 | swine | Included |
| KX377924 | China | Mar-2016 | swine | Included |
| KX751943 | China | Nov-2015 | swine | Included |
| KX751944 | China | Jan-2016 | swine | Included |
| KX751945 | China | Jan-2016 | swine | Included |
| KX751946 | China | Jan-2016 | swine | Included |
| KX778101 | USA | 29-Jul-2015 | swine | Included |
| KX857728 | Colombia | Feb-2016 | porcine | Included |
| KY038016 | China | 11-Sep-2016 | swine | Included |
| KY172968 | USA | 2014-07 | Sus scrofa domesticus | Included |
| KY419132 | China | 2016-11-10 | pigs | Included |
| KY486156 | Canada | 30-Oct-2015 | Not available | Included |
| KY486157 | Canada | 30-Oct-2015 | Not available | Included |
| KY486158 | Canada | 30-Oct-2015 | Not available | Included |
| KY486159 | Canada | 06-Nov-2015 | Not available | Included |
| KY486160 | Canada | 06-Nov-2015 | Not available | Included |
| KY486161 | Canada | 06-Nov-2015 | Not available | Included |
| KY486162 | Canada | 01-Dec-2015 | Not available | Included |
| KY486163 | Canada | 01-Dec-2015 | Not available | Included |
| KY486164 | Canada | 01-Dec-2015 | Not available | Included |
| KY486165 | Canada | 10-Oct-2015 | pig | Included |
| KY486166 | Canada | 10-Oct-2015 | pig | Included |
| KY618834 | USA: Indiana | 09-Oct-2015 | swine | Included |
| KY618835 | USA: Indiana | 01-Jul-2016 | swine | Included |
| KY618836 | USA: Indiana | 05-Aug-2016 | swine | Included |
| KY618837 | USA: Indiana | 16-Sep-2016 | swine | Included |
| KY747510 | China | Jan-2017 | porcine | Included |
| KY747511 | China | Jan-2017 | porcine | Included |
| KY747512 | China | Jan-2017 | porcine | Included |
| MF189000 | China | Mar-2017 | porcine | Included |
| MF189001 | China | Mar-2017 | porcine | Excluded |
| MF460449 | China | Jun-2017 | swine | Included |
| MF615501 | Brazil: SC | Jan-2015 | swine | Included |
| MF615502 | Brazil: SC | May-2016 | swine | Included |
| MF615503 | Brazil: SC | May-2016 | swine | Included |
| MF615504 | Brazil: SC | May-2016 | swine | Included |
| MF615505 | Brazil: SC | May-2016 | swine | Included |
| MF615506 | Brazil: SC | Aug-2015 | swine | Included |
| MF615507 | Brazil: SC | Oct-2015 | swine | Included |
| MF615508 | Brazil: SC | Nov-2015 | swine | Included |
| MF615509 | Brazil: SC | Nov-2015 | swine | Included |
| MF615510 | Brazil: SC | Nov-2015 | swine | Included |
| MF893200 | China | 01-Mar-2016 | swine | Included |
| MF967574 | China | Aug-2017 | swine | Included |
| MG428680 | China | 16-Jun-2017 | porcine | Included |
| MG428681 | China | 17-Jun-2017 | porcine | Included |
| MG428682 | China | 20-Jun-2017 | porcine | Included |
| MG428683 | China | 30-Jun-2017 | porcine | Included |
| MG428684 | China | 15-Jul-2017 | porcine | Included |
| MG428685 | China | 20-Jul-2017 | porcine | Included |
| MG765550 | China | 27-Jun-2017 | swine | Included |
| MG765551 | China | 25-Jul-2017 | swine | Included |
| MG765552 | China | 07-Jul-2017 | swine | Included |
| MG765553 | China | 25-Jun-2017 | swine | Included |
| MG765554 | China | 02-Sep-2017 | swine | Included |
| MG765555 | China | 01-Sep-2017 | swine | Included |
| MG765556 | China | 19-Jul-2017 | swine | Included |
| MG765557 | China | 15-Jun-2017 | swine | Included |
| MG765558 | China | 29-Aug-2017 | swine | Included |
| MG765559 | China | 05-May-2017 | swine | Included |
| MG765560 | China | 03-May-2017 | swine | Included |
| MG765561 | China | 26-Apr-2017 | swine | Included |
| MG765562 | China | 27-Apr-2017 | swine | Included |
| MG765563 | China | 26-Apr-2017 | swine | Included |
| MG765564 | China | 19-Apr-2017 | swine | Included |
| MG765565 | China | 07-Jul-2017 | swine | Included |
| MG765566 | China | 16-Feb-2017 | swine | Included |
| MH064433 | China | Nov-2017 | swine | Included |
| MH064434 | China | Nov-2017 | swine | Included |
| MH064435 | China | Dec-2017 | swine | Included |
| MH064436 | China | Dec-2017 | swine | Excluded |
| MH316113 | China | Sep-2017 | swine | Excluded |
| MH316114 | China | Sep-2017 | swine | Included |
| MH316115 | China | Sep-2017 | swine | Included |
| MH316116 | China | Sep-2017 | swine | Included |
| MH316117 | China | Sep-2017 | swine | Included |
| MH490944 | China | Jul-2017 | swine | Included |
| MH588717 | China | 20-May-2018 | wild boar | Included |
| MH634506 | USA | 2017 | swine | Included |
| MH634507 | USA | 2017 | swine | Included |
| MH634508 | USA | 2017 | swine | Included |
| MH634509 | USA | 2017 | swine | Included |
| MH634510 | USA | 2017 | swine | Included |
| MH634511 | USA | 2017 | swine | Included |
| MH634512 | USA | 2017 | swine | Included |
| MH634513 | USA | 2017 | swine | Included |
| MH634514 | USA | 2017 | swine | Included |
| MH634515 | USA | 2017 | swine | Included |
| MH634516 | USA | 2017 | swine | Included |
| MH634517 | USA | 2017 | swine | Included |
| MH634518 | USA | 2017 | swine | Included |
| MH634519 | USA | 2017 | swine | Included |
| MH634520 | USA | 2017 | swine | Included |
| MH634521 | USA | 2017 | swine | Included |
| MH634522 | USA | 2017 | swine | Included |
| MH634523 | USA | 2015 | swine | Included |
| MH634524 | USA | 2015 | swine | Included |
| MH634525 | USA | 2015 | swine | Included |
| MH634526 | USA | 2015 | swine | Included |
| MH634527 | USA | 2015 | swine | Included |
| MH634528 | USA | 2015 | swine | Included |
| MH634529 | USA | 2015 | swine | Included |
| MH634530 | USA | 2015 | swine | Included |
| MH634531 | USA | 2015 | swine | Included |
| MH634532 | USA | 2015 | swine | Included |
| MH634533 | USA | 2015 | swine | Included |
| MH634534 | USA | 2015 | swine | Included |
| MH704432 | Viet Nam | 2018 | swine | Included |
| MH716015 | China | Jan-2018 | swine | Included |
| MH817445 | China: Sichuan | 20-May-2018 | porcine | Included |
| MH817446 | China: Sichuan | 20-May-2018 | porcine | Included |
| MH844686 | China | Not available | Sus scrofa (swine) | Included |
| MH844687 | China | Not available | Sus scrofa (swine) | Included |
| MH844688 | China | Not available | Sus scrofa (swine) | Included |
| MK039162 | China | Sep-2018 | swine | Included |
| MK170054 | China | 2018 | porcine | Included |
| MK170055 | China | 2018 | porcine | Included |
| MK170056 | China | 2017 | porcine | Included |
| MK252002 | China | 2018 | Bos taurus (cattle) | Included |
| MK256736 | China | 03-Jun-2018 | porcine | Included |
| MK284514 | China | 09-Nov-2018 | porcine | Included |
| MK284515 | China | 16-May-2018 | porcine | Included |
| MK333629 | USA | 31-Aug-2015 | porcine | Included |
| MK333630 | USA | 04-Sep-2015 | porcine | Included |
| MK333631 | USA | 04-Sep-2015 | porcine | Included |
| MK333632 | USA | 30-Sep-2015 | porcine | Included |
| MK333633 | USA | 13-Jul-2016 | porcine | Included |
| MK333634 | USA | 14-Jul-2016 | porcine | Included |
| MK333635 | USA | 14-Jul-2016 | porcine | Included |
| MK333636 | USA | 15-Jul-2016 | porcine | Included |
| MK333637 | USA | 02-Aug-2016 | porcine | Included |
| MK357115 | China | 13-Nov-2018 | pig | Included |
| MK357116 | China | 24-Nov-2018 | pig | Included |
| MK357117 | China | 26-Nov-2018 | pig | Included |
| MK463618 | China | Mar-2018 | pig | Included |
| MK463619 | China | Mar-2018 | pig | Included |
| MK802890 | China | Mar-2018 | pig | Included |
| MK802892 | China | Mar-2018 | pig | Included |
| MN017170 | China | 15-Sep-2018 | swine | Included |
| MN164664 | USA | 2015 | swine | Included |
| MN171528 | Not available | Not available | Not available | Included |
| MN233024 | USA: MO | 15-Sep-2015 | porcine | Included |
| MN233025 | USA: KS | 02-Oct-2015 | porcine | Included |
| MN233026 | USA: NC | Mar-1988 | porcine | Included |
| MN233027 | USA: MN | 05-Jul-1988 | porcine | Included |
| MN233028 | USA: IA | 28-Aug-1989 | porcine | Included |
| MN233029 | USA: NJ | 14-Dec-1989 | porcine | Included |
| MN233030 | USA: IA | 1990 | porcine | Included |
| MN233031 | USA: IL | 16-Sep-1992 | porcine | Included |
| MN233032 | USA: IL | 16-Dec-1993 | porcine | Included |
| MN233033 | USA: MN | 16-Jul-1999 | porcine | Included |
| MN423333 | China | Not available | Sus scrofa | Included |
| MN423334 | China | Not available | Sus scrofa | Included |
| MN433300 | China | Jan-2018 | pig | Included |
| MN615881 | China | Oct-2018 | buffalo | Included |
| MN700930 | China | Jul-2018 | Sus scrofa | Included |
| MN781981 | China | Not available | Sus scrofa | Included |
| MN781982 | China | Not available | Sus scrofa | Included |
| MN781983 | China | Not available | Sus scrofa | Included |
| MN781984 | China | Not available | Sus scrofa | Included |
| MN887248 | China | May-2019 | porcine | Included |
| MN887249 | China | Nov-2019 | porcine | Included |
| MN887250 | China | Aug-2019 | porcine | Included |
| MN887251 | China | Dec-2019 | porcine | Included |
| MN922286 | China | 11-Sep-2017 | swine | Included |
| MT457474 | China | 2019 | swine | Included |
| MT713137 | China | Nov-2019 | Sus scrofa | Included |
| MT840202 | China | 2018 | Sus scrofa | Included |
| MW117126 | China | Mar-2020 | swine | Included |
| MW117127 | China | Mar-2020 | swine | Included |
| MW117128 | China | Mar-2020 | swine | Included |
| MW117129 | China | Mar-2020 | swine | Included |
| MW713116 | China: Guangdong | 2019 | Sus scrofa | Included |
| MW713117 | China: Guangdong | 2019 | Sus scrofa | Included |
| MW713118 | China: Guangdong | 2018 | Sus scrofa | Included |
| MW713119 | China: Guangdong | 2019 | Sus scrofa | Included |
| MW713120 | China: Guangdong | 2018 | Sus scrofa | Included |
| MW713121 | China: Guangdong | 2019 | Sus scrofa | Included |
| MW713122 | China: Guangdong | 2019 | Sus scrofa | Included |
| MW713123 | China: Guangdong | 2019 | Sus scrofa | Included |
| MZ375462 | China | 2019 | Sus scrofa | Included |
| MZ818785 | China | 24-Jun-2017 | pig | Included |
| NC_011349 | USA | Not available | Not available | Included |
| OM654411 | China | Dec-2020/Sep-2021 | swine | Included |
| ON024061 | China: Guangxi Province | Jun-2017 | Sus scrofa | Included |
| ON868368 | China | 01-Mar-2020 | swine | Included |
| ON868369 | China | 01-Mar-2020 | swine | Included |
| ON868370 | China | 01-Sep-2020 | swine | Included |
| ON868371 | China | 01-Sep-2020 | swine | Included |
| ON868372 | China | 01-Apr-2020 | swine | Included |
| ON868373 | China | 01-Sep-2018 | swine | Included |
| ON868374 | China | 01-Mar-2020 | swine | Included |
| ON868375 | China | 01-Sep-2020 | swine | Included |
| ON868376 | China | 01-Dec-2021 | swine | Included |
| ON868377 | China | 01-Dec-2021 | swine | Included |
| OP562896 | China | 15-Feb-2022 | swine | Included |
| OP714192 | China | 04-Jul-2022 | pig | Included |
| OQ162219 | China | 2018 | swine | Included |
| OR416237 | USA: Alaska | 2017 | Phocoena phocoena | Included |
| OR416238 | USA: Alaska | 2017 | Phocoena phocoena | Included |
| OR416239 | USA: Alaska | 2019 | Delphinapterus leucas | Included |
| OR703632 | China | 2020 | swine | Included |
| OR759780 | China | 2023 | Not available | Included |
| PZ899952 | China | 2016 | Sus scrofa | Included |
| PZ899953 | China | 2017 | Sus scrofa | Included |

This table summarizes the GenBank accession numbers and geographical origins (countries/regions) of all representative SVA strains enrolled in this study. “Included” indicates sequences used in the BEAST analyses. MH316113, MF189001, and MH064436 were excluded because they were identified as putative recombinant sequences.
